# Modelling and Simulation of Fuel Cell Dynamics for Electrical Energy Usage of Hercules Airplanes

**DOI:** 10.1155/2014/593121

**Published:** 2014-03-20

**Authors:** Hamid Radmanesh, Seyed Saeid Heidari Yazdi, G. B. Gharehpetian, S. H. Fathi

**Affiliations:** ^1^Electrical Engineering Department, Islamic Azad University, Takestan Branch, Takestan 19585-466, Iran; ^2^Electrical Engineering Department, Amirkabir University of Technology, Tehran 15875-4413, Iran

## Abstract

Dynamics of proton exchange membrane fuel cells (PEMFC) with hydrogen storage system for generating part of Hercules airplanes electrical energy is presented. Feasibility of using fuel cell (FC) for this airplane is evaluated by means of simulations. Temperature change and dual layer capacity effect are considered in all simulations. Using a three-level 3-phase inverter, FC's output voltage is connected to the essential bus of the airplane. Moreover, it is possible to connect FC's output voltage to airplane DC bus alternatively. PID controller is presented to control flow of hydrogen and oxygen to FC and improve transient and steady state responses of the output voltage to load disturbances. FC's output voltage is regulated via an ultracapacitor. Simulations are carried out via MATLAB/SIMULINK and results show that the load tracking and output voltage regulation are acceptable. The proposed system utilizes an electrolyser to generate hydrogen and a tank for storage. Therefore, there is no need for batteries. Moreover, the generated oxygen could be used in other applications in airplane.

## 1. Introduction

In 1956, the US Air Force began development on a Bacon fuel cell for aerospace applications. Development of a regenerative fuel cell that can also be used to electrolyze water into hydrogen and oxygen started during the 1960s. These developments advanced fuel cell technology for the shuttle vehicle and its future upgrades [1]. In the 1980s, the US Air Force developed fuel cell technology for Alaskan remote radar sites [1]. FCs could generate electricity and heat from chemical processes [2–6]. Their applications have increased significantly and they could be implemented in many industries such as microelectronics, small boats, airplanes, bus, and combined heat and power (CHP) applications. NASA improved the space shuttle operations and as a result a program to upgrade the existing fuel cell power plant was begun. The results were replacing the alkaline fuel cell (AFC) with a proton exchange membrane (PEM) fuel cell system, resulting in a much lower life cycle cost of the power plant [2–6]. Japan Agency for Marine-Earth Science and Technology and Mitsubishi Heavy Industries have been developing the autonomous underwater vehicle “Urashima” since 1998. Long distance cruising auks generally need an air independent propulsion power source characterized by high energy density and high energy efficiency. It is understood that one of the main themes of fuel cell is the hydrogen storage; the metal hydride storage has been adopted for “Urashima” as a safer solution for storing the hydrogen [7]. Optimization of fuel cell and supercapacitor for electric vehicles application has been discussed in [8]. A feasibility study for on-board power generation using a combination of solid oxide fuel cells and gas turbines has been presented in [9]. The purpose of this study is to investigate the potential use of fuel-cell-based auxiliary power unit (APUs) for on-board power generation of commercial aircraft [10]. Power control strategies for the propulsion of unmanned aerial vehicle (UAV) which is driven by fuel cell and battery as a hybrid system have been studied in [11–16]. A multiphysical proton exchange membrane fuel cell stack model, which is suitable for real-time emulation, has been presented in [17, 18]. In [19], a DC hybrid power source composed of PEM fuel cell as main source, Li-ion battery storage as transient power source, and their interfacing convertors has been modeled. In [20], the effect of the fuel cell and photovoltaic hybrid system on the distribution network has been studied. For determining the capacity of each distributed generation source, the voltage limitation on bus voltages under different conditions has been considered.

**Figure 1:**
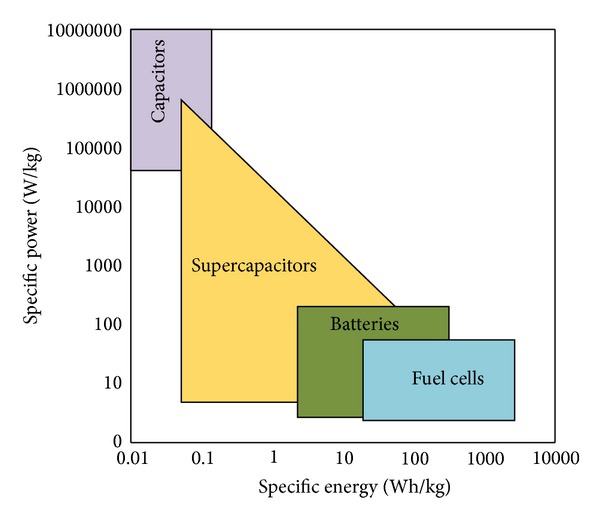
Power and energy density of FC, batteries, and capacitors [1].

As shown in Figure 1, FCs have superior energy densities over batteries and ultracapacitors. Moreover, PEMFC has superior advantages like fast start-up and ability to feed partial loads which make it an appropriate option for generating part of start-up electrical energy of Hercules airplanes. In [21], a new zero voltage switching current-fed DC-DC converter has been presented which has high voltage gain. In this converter, all switches (main and auxiliary) turn on under zero voltage switching and turn off under almost zero voltage switching due to snubber capacitor. If there is an outage in one or two generators, the electrical need of the commercial aircraft is supplied via main generators which is installed on each main engine and also a small AC generator on the Auxiliary Power Unit (APU). In flight, the efficiency of the electric power generated by the main engines and their generators is about 30–40%. While on the ground, the average fuel efficiency of the turbine powered APU is typically less than 20% and also has undesirable noise and emissions [22–25]. This is a challenge for aircraft manufacturers to reduce the fuel consumption while simultaneously reducing emissions. Hence, there is very strong interest in developing fuel cells for aerospace applications. So, this is focused on in fuel cell application in C-130 Hercules aircraft.

## 2. Energy System of C-130 Aircraft

C-130 airplanes have 4 motors connected to generators via gearbox. The generator terminals are connected to distribution system by cable and fuselage as neutral point. This voltage is 115 V line to ground and 200 V line to line and has a frequency of 380 Hz–420 Hz. The fifth generator is a small generator with high speed and for air suction to airplane, which is essential to its start-up. This sensitive generator will generate the energy for emergency cases. It is extremely sensitive to temperature so that its output is half of that at land with respect to height [26].

**Figure 2:**
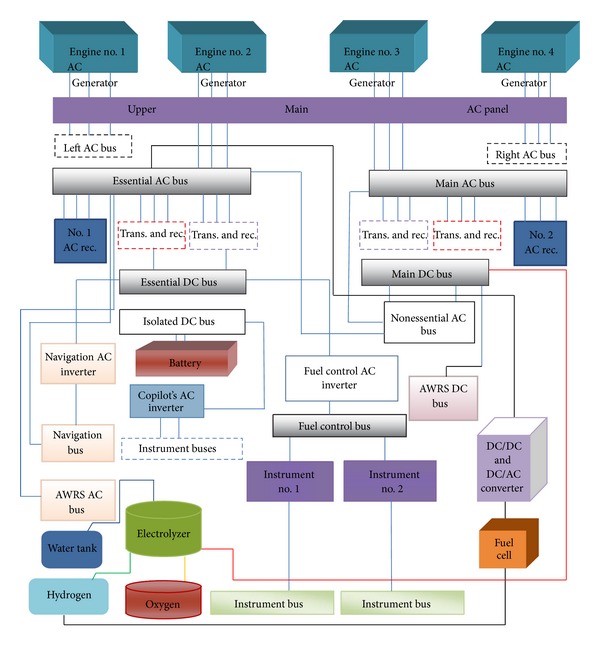
C-130 airplane electrical distribution system connected to FC.

The first 4 generators are 40 kVA and the fifth one acts like a 30 kVA machine on air and like a 20 kVA machine on land. To start the airplane, one could use AC or DC external sources since airplane start-up current is very high. The external source could be a 200 V and 400 Hz AC source or a 28 V and 400 A DC source. This is another external type that its output is high speed wind which passes through the fifth generator in airplane in order to generate electrical energy needed for airplane's start-up. The generated electrical power flows through 4 main, right, left, and essential AC buses. It is used in different parts of airplane via transformer and AC-DC converters. Most of airplane equipment uses DC power, so the airplane distribution system is divided into AC and DC parts as shown in Figure 2.

If one or two generator outage, the remaining generators can generate the needed electrical energy without any problem. But in case of outage of 3 generators, relays will disconnect all buses except the essential bus. In this case, hydraulic systems will fall down and just a few essential systems will continue operating. DC buses are also divided into 4 main, essential, isolated, and battery buses. The main and essential buses are connected via a relay allowing power flow from the main bus to essential one during the flight, while blocking reverse direct power flow. There is the same configuration between essential and isolated buses; however, reverse power flow is allowed on the land. Isolated bus is connected to battery bus via a switch. While airplane is on land, all of its electrical energy is supplied from battery, battery bus, and isolated bus. On the other hand, during start-up, the battery bus is disconnected and essential bus gathers electrical energy from external source and passes it to starter and other parts.

As mentioned before, to start the airplane it is required to use extra sources which are massive, consuming, and expensive and usually are found in standard airports where C-130 can land. C-130 airplane can land on terrestrial band where there is no external source. Therefore, airplane cannot fly again. There is a solution that one could carry the external source and staff whose cost is high and reduce airplanes efficiency. Moreover, loss of 3 generators is a dangerous case. So it is required to equip airplane with an essential electricity system to enhance reliability. The proposed system includes the mentioned FCs which is connected to DC essential bus via a DC-DC converter or to AC essential bus via a DC-AC converter.

Novel FC system could successfully decrease the Hercules problems which are mentioned above.

## 3. System Components

The proposed system includes 65 PEMFCs, electrolyser, DC-DC and DC-AC converters, ultracapacitor, and multiple controllers. The FC system, that is, 65 individual FCs in series and their output current, can be between 0 A and 5 A. The hydrogen and oxygen flow rate is controlled via PID controllers to regulate the system output voltage at 48V. In this paper, a system including electrolyser and hydrogen tank is used to generate electrical energy needed for airplane's motors start-up. This system will replace the airplanes external power supply which has disadvantages such as high cost, maintenance problem, and high failure rates. Moreover, the proposed system could be used as a reserve power supply.

An electrolyser is used for generating FC's hydrogen, which operates in two main modes.

1. *Active motor.* The motor of airplane is ON with light load and a part of its energy is transferred to the electrolyser to generate hydrogen and oxygen for the use in the next mode.
2. *Airplane Start-Up.* The stored hydrogen and oxygen are transferred to FC in order to generate electrical energy for airplane start-up.

### 3.1. FC Model

The used FC is from a PEMFC which includes a single layer electrolyte in contact with anode and cathode. There are several ways to model PEMFC.

The FC Nernst voltage is equal to 1.22 V with H_2_O generated [27]. However, the actual voltage of FC is less than the ideal voltage due to irreversible losses in FC system. *E*_Nernst_ is calculated as follows [28]:

(1) ENernst=1.2209−0.85×10−3(T−2098.15) +4.3085×10−3×T×(ln⁡PH2+0.5ln⁡PO2),

where *P* and *T* represent the effective pressure and temperature, respectively. Undissolved oxygen concentration in gas/liquid intermediary could be calculated using Henry law as below:

(2) $$\begin{array}{c} C_{{\text{O}}_{2}}=\frac{P_{{\text{O}}_{2}}}{5.08\times 10^{6}\times \exp\left(-498/T\right)}. \end{array}$$

Overvoltages due to internal process and resistance are calculated from an experimental equation:

(3) $$\begin{array}{c} \eta_{\text{act}}=-0.9514+0.003120T\times \ln\left(i\right)+7.4\times 10^{-5}T\times \ln\left(C_{{\text{O}}_{2}}\right), \end{array}$$

(4) $$\begin{array}{c} R_{\text{in}}=0.01605-3.5\times 10^{-5}T+8\times 10^{-5}i. \end{array}$$

In (4), *i* is the current flowing in FC and stir resistance is

(5) $$\begin{array}{c} R_{a}=-\frac{\eta_{\text{act}}}{i}. \end{array}$$

Coordinated thermodynamic, mass transfer, and kinetic energy effect determine FC's output voltage:

(6) $$\begin{array}{c} V=E-v_{\text{act}}+\eta_{\text{ohmic}}. \end{array}$$

The voltage drop in FC is compensated by increasing FC's pressure. The dynamic response of a FC could be analysed by adding a capacitor to steady state model. Double layer charge effect is considered by adding a parallel capacitor in model. The differential equation describing FC's voltage is as follows:

(7) $$\begin{array}{c} \frac{dv_{\text{act}}}{dt}=\frac{i}{C}-\frac{v_{\text{act}}}{R_{a}\times C}. \end{array}$$

And the ohmic voltage drop is as follows:

(8) $$\begin{array}{c} \eta_{\text{ohmic}}=-i\times R_{\text{in}}. \end{array}$$

The proposed FC includes 65 series cells. So its output voltage is equal to

(9) $$\begin{array}{c} V_{\text{stack}}=130V_{\text{cell}}. \end{array}$$

O_2_ and H_2_ consumption in FC depends on input/output rate and FC's current. Using input/output rate (mol/s), one can calculate the pressure of the gas (in FC's humidifier) using the mol's equality law.

For FC's anode,

(10) $$\begin{array}{c} \frac{V_{a}}{RT}\cdot \frac{dP_{{\text{H}}_{2}}}{dt}=m\rho_{{\text{H}}_{2}}-{\left(\rho_{{\text{H}}_{2}}\cdot U\cdot A\right)}_{\text{out}}-\frac{i}{2F}. \end{array}$$

Similarly, for FC's cathode,

(11) $$\begin{array}{c} \frac{V_{C}}{RT}\cdot \frac{dP_{{\text{O}}_{2}}}{dt}=m\rho_{{\text{O}}_{2\text{in}}}-{\left(\rho_{{\text{O}}_{2}}\cdot U\cdot A\right)}_{\text{out}}-\frac{i}{4F}. \end{array}$$

The used symbols in (1)–(11) are defined in Table 1.

In this paper, the anode and cathode volume is assumed to be 2L. The total balance of the thermal energy in a FC cooled by air can be written as follows:

(12) $$\begin{array}{c} Q_{l}=Q_{s}+Q_{L}, \end{array}$$

where *Q*_*l*_, *Q*_*s*_, and *Q*_*L*_ represent generated, stored, and internal dissipated heat, respectively. To calculate internal lossed heat, FC's current and internal resistance are used as below for 130 cells:

(13) $$\begin{array}{c} \text{internal}\;\text{generated}\;\text{temperature}=i^{2}\left(R_{a}+R_{\mathrm{int}}\right)\times 130. \end{array}$$

The stored thermal energy in FC is calculated by the following equation:

(14) $$\begin{array}{c} \text{stored}\;\text{thermal}\;\text{energy}=C_{t}\times \frac{dT}{dt}, \end{array}$$

where *C*_*t*_ and *T* represent the heat capacity (equal to 100  J/C) and FC's temperature, respectively. Consider

(15) $$\begin{array}{c} \text{thermal}\;\text{loss}\;\text{power}\;\text{to}\;\text{ambient}=\frac{\left(T-T_{a}\right)}{R_{t}}. \end{array}$$

Substituting (13)–(15) into (12) results in

(16) $$\begin{array}{c} \frac{dT}{dt}=\frac{130\times \left(R_{a}+R_{\mathrm{int}}\right)\times i^{2}}{C_{t}}-\frac{\left(T-T_{a}\right)}{R_{t}\times C_{t}}. \end{array}$$

In (16), *T*_*a*_ and *R*_*t*_'s values are 25°C and 0.04°C/W [29], respectively. Equations (1)–(16) represent FC's dynamic behaviour neglecting the dynamic effect of compressors and valves.

### 3.2. Ultracapacitor Model

**Figure 3:**
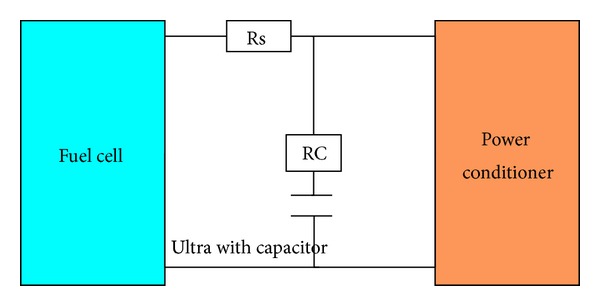
Capacitor bank in parallel with FC.

An ultracapacitor is an energy storage device similar to conventional batteries. These capacitors include two electrodes floating in an electrolyte and they are separated via an isolator. Electrodes are constructed from a porous material. Cross section of these capacitor's electrodes is 500–2000 m^2^/g, that is, larger than the same used in batteries [30, 31]. Capacitor banks with 42 V or higher order voltages could be built using these capacitors. Modules with higher capacitance and voltage ratings could be built using large capacitor banks connected in series and setting an active/reactive power balancing cell. A 435 F and 14 V capacitor module is modelled for the proposed system. To reach 42 V voltage, one can use 5 modules in series. The selected module has 4 mΩ series resistance and 10 mA leakage current. In simulations, the leakage current is assumed to be constant and the current needed for cooling the system is neglected. Therefore, the capacitor module is modelled by a capacitor in series with a resistance, with 108.75 F capacitance and 16 mΩ resistance as shown in Figure 3.

The ultracapacitor is modelled like a low pass filter (LPF) by the following transfer function [32]:

(17) $$\begin{array}{c} \frac{V_{\text{ucap}}}{V_{\text{stack}}}=\frac{s+1/\left(R_{s}\cdot C\right)}{s\left(1+R_{s}/R_{c}\right)+1/\left(R_{s}\cdot C\right)}. \end{array}$$

### 3.3. Electrolyser Dynamic Model

An electrolyser system includes several electrolyser cells connected in series. Their *V*-*I* characteristic depends on temperature and usually it is extremely nonlinear and could be obtained by curve fitting. According to Faraday's law, the rate of generating H_2_ in electrolyser is proportional to the rate of current flowing in electrodes, which is actually the current in output circuit [33, 34]:

(18) $$\begin{array}{c} \eta_{{\text{H}}_{2}}=\frac{\eta_{F}\cdot n_{n}\cdot i_{e}}{2F\;\left(\text{mol}/\text{s}\right)}. \end{array}$$

In (18), *i*_*e*_, *n*_*n*_, and *η*_*F*_ represent electrolyser's current, number of series electrolysers, and Faraday's efficiency, respectively. Faraday's efficiency is the ratio of the maximum practical generated H_2_ to maximum theoretical possible generation of H_2_. Assuming the operation temperature of 40°C, it is equal to

(19) $$\begin{array}{c} \eta_{F}=96.5\times \exp\left(\frac{0.09}{i_{e}}-\frac{75.5}{i_{e}^{2}}\right). \end{array}$$

**Figure 4:**
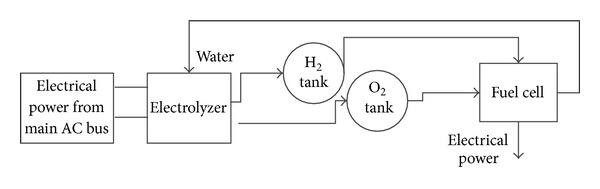
FC, hydrogen tank, oxygen tank, and electrolyser.

Equations (18) and (19) represent a simple model of electrolyser assuming that FC has an autonomous cooling system for regulating the temperature at 40°C.  Figure 4 shows a schematic of a FC connected to electrolyser while the FC's electrical energy is supplied by airplane's essential AC bus.

### 3.4. DC-DC Converter Model

**Figure 5:**
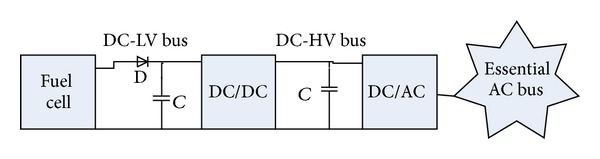
FC connection to AC bus.

In C-130 airplanes, the converter module shown in Figure 5 has two stages for voltage and frequency regulation. First stage is a DC-DC boost converter which regulates the output voltage at a high constant DC voltage while its input is a low varying voltage.

This boost converter is controlled by a PID controller in order to regulate the output voltage at 200 V. This can be achieved by proper tuning of *D* (duty cycle) in the following equation:

(20) $$\begin{array}{c} \frac{V_{\text{boost}}}{V_{\text{ucap}}}=\frac{1}{1-D}. \end{array}$$

**Figure 6:**
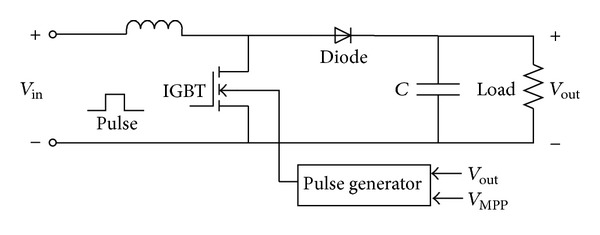
Boost converter.

Figure 6 shows the converter, its pulse generator, and the controller.

To determine the values of the convertor inductor and capacitor, one can use the following equations [35, 36]:

(21) $$\begin{array}{c} L_{C}=\frac{k\left(1-k\right)\cdot R}{2f},\\ C_{C}=\frac{V_{C}\cdot k}{f\cdot R\cdot \Delta V_{c}}, \end{array}$$

whose symbols are defined in Table 2.

So, one can calculate proper inductance and capacitance as below:

(22) LC=k(1−k)R2f=0.4204×(1−0.4204)×902×10000=1.1×10−3H,CC=VC·kf·R·ΔVC=625  ×  0.4410000×90×5.6=54×10−6F.

The DC-DC converter pulse generator should satisfy these following duties:

- maximum power point tracking (MPPT),
- boosting voltage up to desired level.

It is possible to change the inverter output voltage by varying DC bus voltage level. Therefore, for known inverter output voltage level, it is possible to tune DC bus voltage in order to have constant output voltage. In this paper, the inverter output voltage is 220*V*_rms_. Therefore, DC bus voltage should be higher than *V*_*ab*1_; that is,

(23) $$\begin{array}{c} V_{ab1}=\frac{4}{\pi }\sqrt{3}\frac{V_{S}}{2}, \end{array}$$

where *V*_*S*_ and *V*_*ab*1_ represent the DC bus voltage and rms value of the fundamental line voltage.

**Figure 7:**
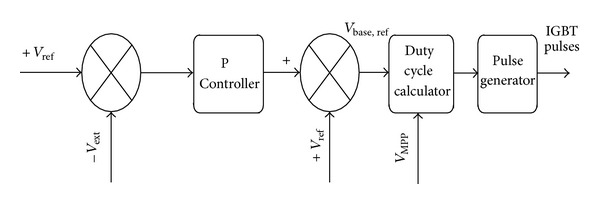
Generating of pulses for switches and their control blocks.

**Figure 8:**
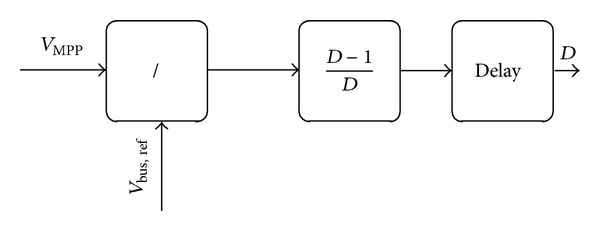
Duty cycle calculator.

As shown in Figure 7, the DC bus voltage is compared to reference 200 V and their discrepancy is passed to a *P* controller in order to be amplified. Afterwards, to have a 200 V output voltage, the resulting error is added to the reference 200 V signal and results in *V*_*dS*_ signal.  Figure 8 shows the procedure of the duty cycle calculator. In this figure, the *V*_bus,ref_ signal is divided into *V*_MPP_ to determine the duty cycle. Moreover, there are solutions in that block to prevent exceeding [0-1] range and fast variations. The resulting duty cycle passes to pulse generator which should generate signals for IGBT switches.

**Figure 9:**
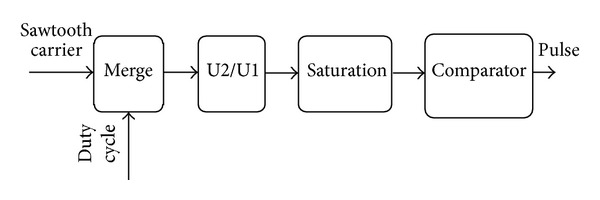
Pulse generator.

Figure 9 shows its internal function.

PWM technique is implemented to generate pulses. Sawtooth carrier frequency is 10 kHz. Afterwards, the generated pulse is used to control DC-DC converter. These two blocks—duty cycle calculator and pulse generator—act as actuator in this closed loop control system.

### 3.5. Inverter Model

**Figure 10:**
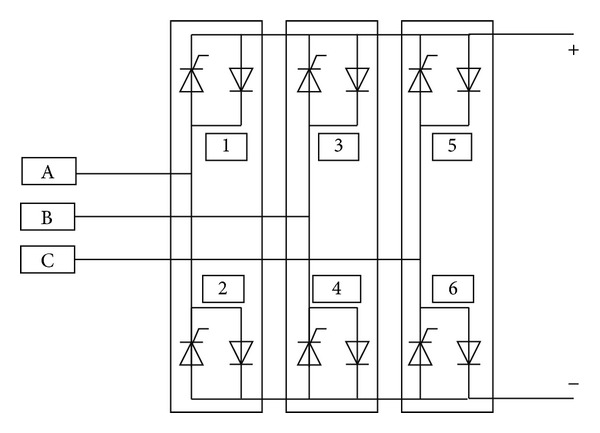
Three-phase inverter.

Figure 10 shows the inverter used in this paper.

The inverter uses a PWM technique with triangle carrier signal frequency of 8 kHz. Regarding circuit properties, medium power rate, high frequency switching, and high input voltage, a three-level converter is used with IGBT switches.

The inverter voltage has large harmonic contents that should be eliminated. According to IEEE standard 519.1992, the THD voltage should be less than 5% and usually needs appropriate use of filters. Since voltages are sinusoidal, odd harmonics are important, so the 3rd–9th order harmonics, 11th–15th harmonics, and 17th–21th harmonics should be less than 4%, 2%, and 1.5%, respectively.

**Figure 11:**
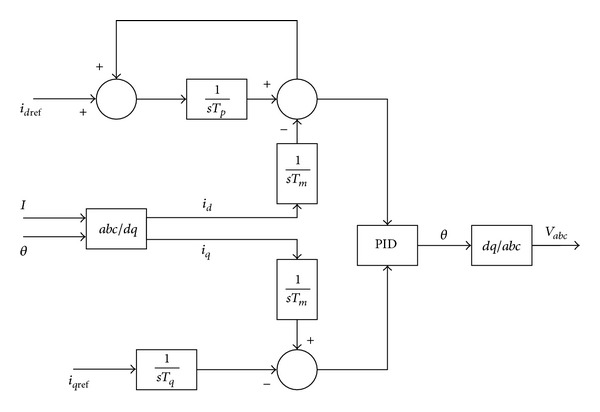
Inverter control system.

To control the current, a close loop current controller as shown in Figure 11 is used.

**Figure 12:**
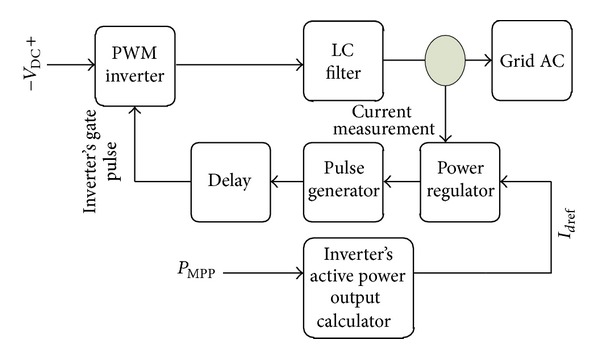
Inverter and its controllers connected to network.

This control system receives *I*_*d*ref_ and *I*_*q*ref_ from an outer control loop and compares them with actual values. As shown in Figure 12, the actual inverter current is measured and transferred to *dq*0 frame via a PLL. One could change active/reactive power with proper setting of *I*_*d*ref_ and *I*_*q*ref_ while *I*_*q*ref_ is usually set to zero. Moreover, there is a closed loop system which adds the *I*_*d*,error_ to *I*_*d*ref_. *I*_*d*ref_ and *I*_*q*ref_ are passed to comparator from a compensator block which naturally is exponential and it is compared to the actual value there. Finally, the error signal is passed to PID controller and passes to *dq*-*ab* *c* transformation block to generate the reference signal for PWM switching. Therefore, PWM switching technique controls the inverter output voltage in order to generate the desired power. Figure 12 shows the inverter schematic and its controllers connected to airplanes electric system.

The main block in this figure is the inverter output active power calculator which sets *I*_*d*ref_ regarding maximum power point as an input. Due to nonlinear nature of system, slow response of system to load changes, and substantial steady state error, there is essential need for a PID controller:

(24) $$\begin{array}{c} G_{r}\left(s\right)=\left(s+T_{d}s^{2}+\frac{1}{T_{i}}\right)\frac{k_{D}}{s}. \end{array}$$

This controller controls its output voltage with varying the input hydrogen and oxygen flow. Also, limiter is used to limit the gas pressure in FC. Ziegler-Nichols solution is used to set PID parameters. Controller parameters are listed in Table 3.

### 3.6. Hydrogen Tank

Storing high pressure H_2_ is the cheapest way to hold it. Although due to advances in composite materials there are 800 atm storage tanks, H_2_ is usually stored in 200 atm–300 atm pressure in steel tanks. In this paper, H_2_ is stored as high pressure gas.

## 4. Simulation Results

The model is simulated by MATLAB/SIMULINK. The simulation model includes 8 subsystems: FC, electrolyser, ultracapacitor, inverter, booster, H_2_ storage, and H_2_ and O_2_ rate of low controller. Each subsystem is a model containing its mathematical equations. A 3-phase inverter is used after DC-DC converter to connect it to AC bus.

**Figure 13:**
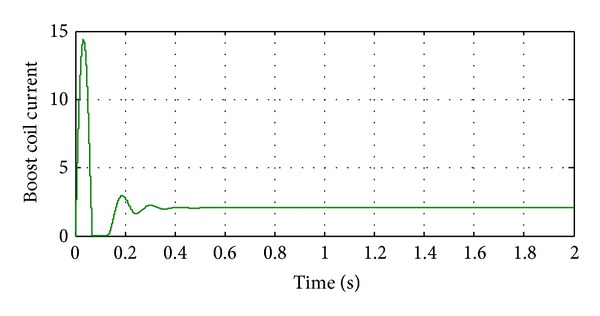
Current of DC-DC converter's inductance.

**Figure 14:**
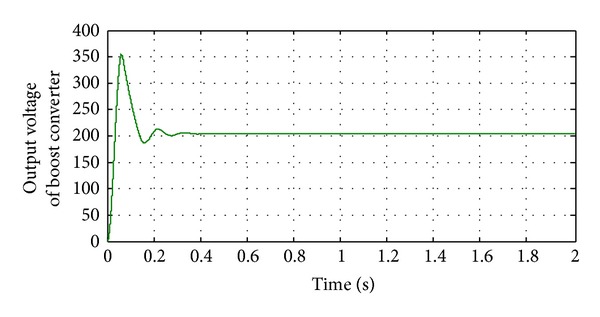
Output voltage of DC-DC converter without implementing filters.

**Figure 15:**
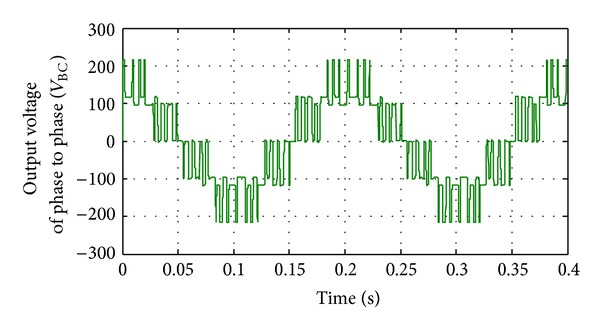
Line to line (B-C) voltage of inverter without implementing filters.

**Figure 16:**
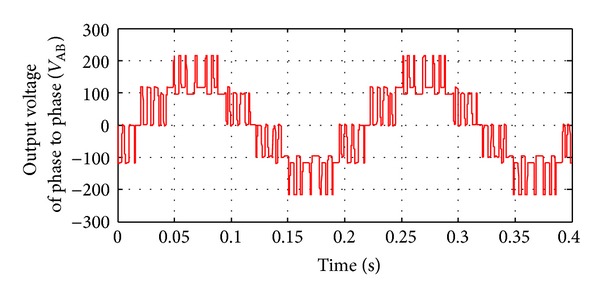
Line to line (A-B) voltage of inverter without implementing filters.

**Figure 17:**
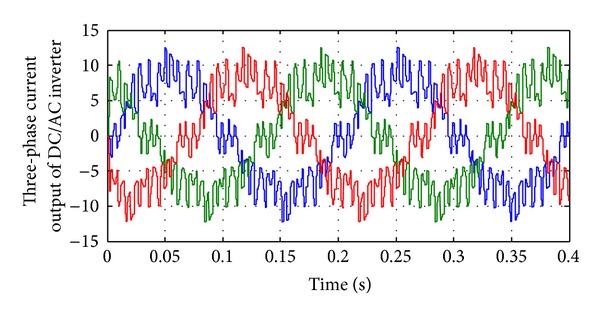
Inverter's output current in worst case THD.

**Figure 18:**
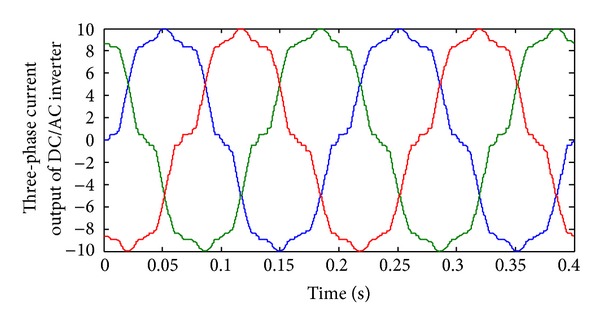
Inverter's output current in best case THD.

Figures 13, 14, 15, 16, 17, and 18 show DC-DC converter's output voltage, filtered voltage, and applied voltage to load and load current.  Figure 17 shows the inverter current for worst case circumstance. Moreover, Figure 18 shows the Inverter's 3-phase output current for best THD circumstance. As shown in Figure 17, current injected to system is low. Using fuel cell in C-130 Hercules as an electrical backup system improves the reliability of electrical system and based on the fly ability is increased. The FC system could generate the oxygen usage for flight personnel. FC application in Hercules causes better flight performance and external generator can be removed accordingly. In emergency cases, FC installation can help the pilot in hydraulic and electric failures. Simulation results successfully show ability of the proposed system in Hercules application.

## 5. Conclusion

In this paper, a FC system was analysed by modelling its dynamic behaviour in C-130 airplane. Then its ability to generate part of airplane needed electrical energy is verified. Simulation results show successful operation of FC systems in aerospace applications without imposing additional costs. Using this system results in the flight reliability improvement. So, one can conclude that hybrid energy generation systems are the best case for islanded networks such as airplane's network. FC system is proposed for islanded operation of airplane networks. Dynamic simulation and modeling are done in MATLAB/SIMULINK and results showed satisfactory results.

## Figures and Tables

**Table 1:** Definitions of the symbols used in (1)–(11).

*m*	Rate of molar flux to humidifier
*V*_*a*_	Anodes volume (L)
*R*	Ideal gas constant, −0.008201 atom/mol·k
*T*	FC's temperature (K)
*V*_*C*_	Anodes volume (L)
*ρ*	Molar density

**Table 2:** Definitions of the symbols used in (21).

Symbols and their definitions	
*L*_*C*_	Critical inductance
*k*	Duty cycle
*R*	Load resistance
*f*	Frequency of switching
*V*_*C*_, Δ*V*_*C*_	Output voltage and its ripple
*C*_*C*_	Critical capacitance

**Table 3:** Controller parameters for oxygen and hydrogen rate controllers.

H_2_ rate	O_2_ rate	Constants
5.00	2.17	*K*_*p*_
0.5	0.5	*T*_*i*_
0	0	*T*_*d*_
